# Veterinary macrolides against *Streptococcus agalactiae*: antibacterial basis, resistance determinants, and PK/PD implications

**DOI:** 10.3389/fvets.2026.1859132

**Published:** 2026-06-22

**Authors:** Huan Liu, Miao An, Na Yu, Yaoxin Tang, Junhao Xiang, Jing Qu, Yiming Liu, Xiubo Li

**Affiliations:** 1 National Feed Drug Reference Laboratories, Feed Research Institute, Chinese Academy of Agricultural Sciences, Beijing, China; 2 Key Laboratory of Animal Antimicrobial Resistance Surveillance, Ministry of Agriculture and Rural Affairs, Feed Research Institute, Chinese Academy of Agricultural Sciences, Beijing, China; 3 Laboratory of Quality and Safety Risk Assessment for Products on Feed-Origin Risk Factor, Ministry of Agriculture and Rural Affairs, Feed Research Institute, Chinese Academy of Agricultural Sciences, Beijing, China

**Keywords:** bovine mastitis, erm(B), PK/PD, resistance determinants, *Streptococcus agalactiae*, veterinary macrolides

## Abstract

*Streptococcus agalactiae* remains an important pathogen causing bovine mastitis, with measurable impacts on herd productivity and milk quality. Veterinary macrolides retain antibacterial activity against a substantial proportion of *S. agalactiae* isolates, as evidenced by *in vitro* susceptibility data across multiple geographic regions, although resistance determinants such as *erm(B)* are prevalent. Pharmacokinetic data indicate that macrolides can achieve significant mammary-site exposure, including prolonged milk persistence and favorable tissue distribution, providing a pharmacological basis for potential efficacy. However, the interpretation of susceptibility is limited by the lack of validated mastitis-specific breakpoints, variable genotype–phenotype concordance, regulatory restrictions, milk residue concerns, and insufficient pathogen-specific clinical outcome data. This review integrates the antibacterial basis, resistance determinants, susceptibility interpretation, regulatory constraints, mammary-site pharmacokinetic/pharmacodynamic (PK/PD) considerations, biofilm potential, and antimicrobial stewardship implications for veterinary macrolides against bovine *S. agalactiae*. Overall, macrolides demonstrate biological plausibility and partial microbiological support, but their clinical utility in treating bovine mastitis remains incompletely defined. Further studies integrating mastitis-specific PK/PD targets, pathogen-stratified clinical outcomes, and longitudinal resistance surveillance are warranted to better define the practical role of veterinary macrolides in bovine *S. agalactiae* mastitis.

## 1 Introduction

Bovine mastitis remains one of the most important infectious diseases in dairy production, and *S. agalactiae* is still a pathogen that cannot be treated as merely a historical concern (1–3). Recent studies continue to recover this organism from both clinical and subclinical mastitis cases in modern dairy systems. In central Thailand, Wataradee et al. (4) characterized 100 *S. agalactiae* isolates from 13 dairy herds and showed that genetically related strains could persist within farms for months. In north China, Liu et al. (5) analyzed 140 isolates collected from clinical mastitis cases on large dairy farms, again confirming that *S. agalactiae* remains epidemiologically important in contemporary herd conditions. These newer datasets fit well with the earlier nationwide Chinese survey by Lin et al. (6), which also documented the continued presence of *S. agalactiae* in dairy cattle with mastitis.

At the same time, the epidemiological picture is no longer as simple as the classical model once suggested. *S. agalactiae* is still recognized as a contagious mastitis pathogen, but field studies indicate that its persistence may involve more than straightforward cow-to-cow transmission during milking. Cobo-Ángel et al. (7) detected *S. agalactiae* not only in milk, but also in rectal and environmental samples in Colombian dairy herds, pointing to possible extramammary reservoirs. Svennesen et al. (8) likewise reported detection on teat skin in infected Danish herds. These observations do not negate the classical contagious paradigm, but they do suggest that herd persistence and re-emergence may be influenced by a broader ecological context (9–11).

Recent antimicrobial surveillance has added another layer of complexity. Across mastitis pathogens isolated from large Chinese dairy farms, Song et al. (12) found that resistance to several commonly used antimicrobials varied substantially by pathogen, while *S. agalactiae* generally retained low resistance to drugs such as amoxicillin/clavulanate, cephalexin, ceftiofur, and rifaximin. More specifically, Liu et al. (5) reported that bovine *S. agalactiae* isolates from north China were most resistant to tetracyclines, followed by macrolides and lincosamides; nevertheless, 89.3% of the 140 isolates remained susceptible to erythromycin, while *erm(B)* was detected in 75.0% of isolates and represented the predominant macrolide resistance determinant. In contrast, studies from Thailand painted a somewhat different picture. Wataradee et al. (4) found that most isolates were susceptible to erythromycin, and Wataradee et al. (13) further reported that 99% of 100 bovine isolates remained erythromycin-susceptible, although multidrug-resistant isolates were also present. Taken together, these findings suggest that the therapeutic profile of bovine *S. agalactiae* is not fixed. It is region-dependent and should be interpreted isolate by isolate rather than assumed from older generalizations (14–18).

Against this background, veterinary macrolides remain of considerable research interest. Their relevance is supported by several factors. First, at the microbiological level, susceptible *S. agalactiae* isolates may still respond to macrolide exposure. Second, at the pharmacological level, many veterinary macrolides are characterized by extensive tissue distribution and prolonged persistence, features that have supported their clinical use in food-producing animals. In cattle, Huang et al. (19) showed that gamithromycin had a large volume of distribution and high bioavailability after subcutaneous administration, and that exposure in lung tissue was markedly higher than in plasma. These pharmacokinetic properties make this class particularly attractive, although the available data have largely been derived from non-mastitis studies (20, 21).

However, the strength of the microbiological evidence is not uniform. Entorf et al. (22) evaluated the susceptibility of 303 streptococcal isolates from bovine mastitis, including 101 *S. agalactiae* isolates, to erythromycin and tylosin, and found that resistant isolates commonly carried macrolide resistance determinants, particularly *erm(B)*. This study not only supports the relevance of veterinary macrolides to bovine *S. agalactiae*, but also highlights an important limitation: antibacterial activity cannot be assumed in the presence of resistance. Likewise, clinical studies on long-acting macrolides in dairy cattle, such as those involving dry-cow therapy, provide useful context, but they have not established clear evidence base specifically for *S. agalactiae* mastitis (23).

A focused reassessment is therefore warranted. The key issue is not whether macrolides can inhibit Gram-positive bacteria in principle, but rather how strong the available evidence is for *S. agalactiae* itself, which resistance determinants are currently most important in bovine isolates, and how existing PK/PD knowledge should be interpreted in a mammary infection-site context. These questions are closely connected, yet they are often discussed in isolation. Accordingly, this review systematically re-examines the activity of veterinary macrolides against *S. agalactiae* from three interrelated perspectives: antibacterial basis, resistance mechanisms, and PK/PD implications.

Throughout this review, three levels of evidence are distinguished. Microbiological plausibility refers to *in vitro* activity against susceptible *S. agalactiae* isolates. Pharmacological plausibility refers to the ability of a macrolide to reach mammary-related compartments at potentially relevant concentrations. Proven clinical efficacy requires pathogen-specific bacteriological or clinical cure data under bovine mastitis conditions. This distinction is essential because the first two levels of evidence do not automatically establish the third.

## 2 Search strategy, eligibility criteria, and evidence appraisal

### 2.1 Search strategy

This review was designed as a critical narrative review rather than a systematic review or meta-analysis. To improve transparency and reproducibility, a structured literature search was conducted in PubMed, Web of Science, Scopus, and Google Scholar. The final search was updated on 9 January 2026. Search terms included combinations of “*S. agalactiae*,” “group B Streptococcus (GBS),” “bovine mastitis,” “veterinary macrolides,” “tylosin,” “tilmicosin,” “tylvalosin,” “tulathromycin,” “gamithromycin,” “tildipirosin,” “macrolide resistance,” “*erm*,” “*mef*,” “*msr*,” “macrolide–lincosamide–streptogramin B (MLSB),” “milk pharmacokinetics,” “PK/PD,” “veterinary breakpoints,” “milk withdrawal,” “biofilm,” and “antimicrobial stewardship.” Reference lists of relevant reviews and key original studies were also screened manually. Regulatory and interpretive documents from organizations such as CLSI, EUCAST/VetCAST, EMA, and FDA were consulted when discussing susceptibility interpretation, residues, withdrawal periods, and extra-label use.

### 2.2 Inclusion and exclusion criteria

Eligible publications included original studies, surveillance reports, pharmacological studies, clinical or dry-cow therapy studies, regulatory documents, interpretive standard documents, and relevant reviews. Priority was given to studies involving bovine *S. agalactiae* isolates from mastitis cases, studies reporting macrolide or MLSB-related susceptibility, studies describing resistance determinants such as *erm*, *mef*, *msr*, *lnu*, *lsa*, *tet(M)*, or *tet(O)*, and pharmacokinetic or PK/PD studies reporting milk, mammary secretion, mammary tissue, somatic cell, or other infection-site-related exposure. Studies on other bovine mastitis streptococci or non-mammary models were included only when direct *S. agalactiae* mastitis data were limited and when they provided mechanistic or pharmacological context.

Studies were excluded when they were unrelated to *S. agalactiae* or bovine mastitis, dealt only with human neonatal or obstetric group B streptococcal disease without mechanistic relevance, lacked identifiable antimicrobial, pathogen, host, or sampling context, or reported only conference abstracts without sufficient methodological or quantitative detail. When duplicate or overlapping datasets were encountered, the most complete or most recent report was prioritized.

### 2.3 Study selection and evidence prioritization

Evidence was prioritized according to its direct relevance to the review question. The highest priority was assigned to studies involving bovine *S. agalactiae* isolates from mastitis cases, especially those reporting minimum inhibitory concentration (MIC) values, resistance phenotypes, resistance genes, or pathogen-specific treatment outcomes. Studies involving other bovine mastitis streptococci were used as supportive evidence when *S. agalactiae*-specific data were unavailable. Pharmacokinetic studies were prioritized when they reported concentrations in milk, mammary secretions, mammary tissue, somatic cells, or other compartments directly related to intramammary infection. Respiratory or non-mammary PK/PD studies were included only to define class-level pharmacological principles and were not treated as direct evidence of efficacy in bovine mastitis.

### 2.4 Quality appraisal of key evidence

Key surveillance studies were appraised qualitatively according to sample size, geographic coverage, isolate origin, bacterial identification method, susceptibility testing method, use of standardized interpretive criteria, and whether resistance genotypes were linked to phenotypes. PK and PK/PD studies were appraised according to animal species, physiological status, administration route, sampling matrix, analytical method, reporting of total versus free drug concentration, and relevance to mastitis conditions. Clinical and dry-cow studies were interpreted cautiously when they enrolled mixed-pathogen infections, lacked pathogen-specific cure rates, or did not stratify outcomes for *S. agalactiae*. Because this review was not intended as a formal systematic review, the following flow diagram is adapted for transparency rather than presented as a PRISMA-compliant quantitative selection record.

**Figure 1:**
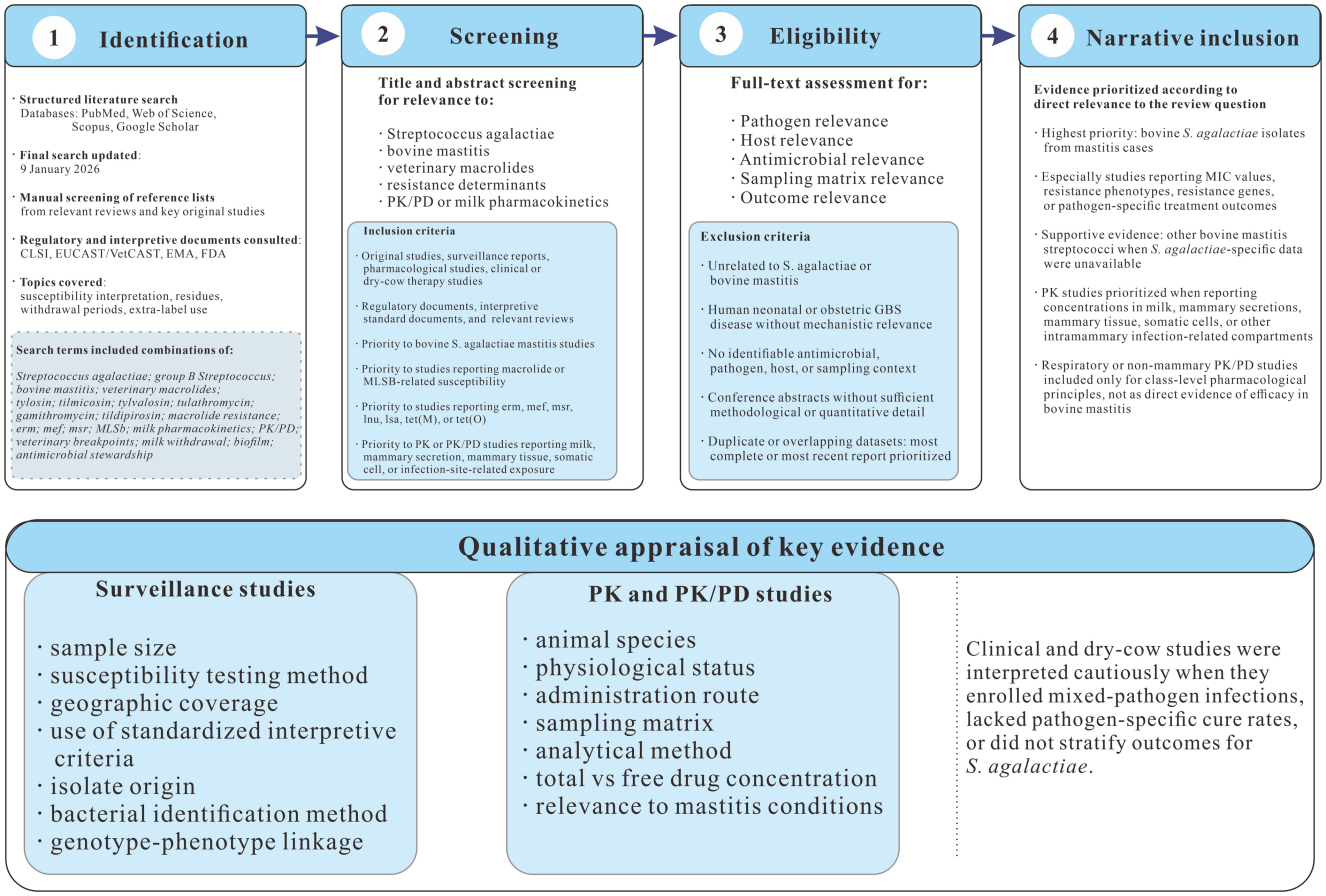
Adapted flow diagram of literature identification and evidence selection. This flow diagram is adapted for transparency in a critical narrative review and is not intended as a PRISMA-compliant quantitative selection record.

Identification (database searches and manual reference screening) → Screening (title and abstract relevance to *S. agalactiae*, bovine mastitis, macrolides, resistance, or PK/PD) → Eligibility (full-text assessment for pathogen, host, antimicrobial, sampling matrix, and outcome relevance) → Narrative inclusion (direct bovine *S. agalactiae* evidence prioritized; indirect evidence used only for mechanistic, regulatory, or pharmacological context; see Figure 1).

## 3 Veterinary macrolides relevant to *S. agalactiae*

**Figure 2:**
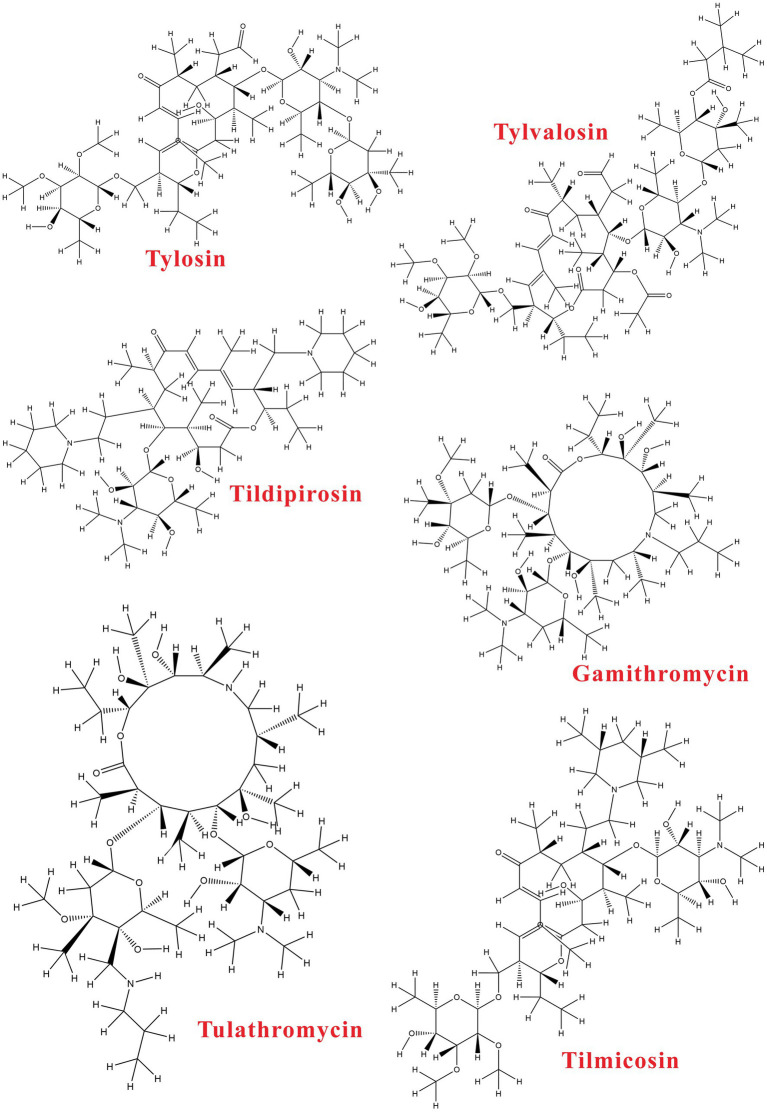
Structural relationship among tylosin-related veterinary macrolides.

The veterinary macrolides discussed in this review do not constitute a completely uniform group. Tylosin, tilmicosin, and tylvalosin (Figure 2) form the core 16-membered macrolide group, with tylosin as the natural prototype and the latter two as semisynthetic derivatives of tylosin (24). These agents are particularly important in food-producing animals because of their activity against Gram-positive bacteria and mycoplasmas, and they are often discussed together because of their structural relatedness. Tildipirosin can be placed close to this core group. It is not a tulathromycin- or gamithromycin-type compound, but rather a semisynthetic derivative of tylosin (25, 26). It is therefore regarded here as a tylosin-related veterinary macrolide, although its use is mainly characterized by its long-acting injectable profile. Tulathromycin and gamithromycin are different. Tulathromycin is a triamilide, whereas gamithromycin is an azalide; both have been used primarily as long-acting veterinary macrolides for bovine respiratory disease (19, 27, 28). Although they fall within the same broader class, they are not derived from the tylosin lineage.

The six macrolides were selected because they represent the main veterinary macrolide categories relevant to this review and because they provide different levels of evidence for bovine *S. agalactiae* mastitis. Tylosin provides the clearest direct microbiological link, as tylosin susceptibility has been compared with erythromycin susceptibility in bovine mastitis streptococci, including *S. agalactiae* isolates (22). Tilmicosin, tylvalosin, and tildipirosin are included as tylosin-related veterinary macrolides because of their structural relationship, Gram-positive antibacterial relevance, and available mammary or milk exposure data, although their evidence for bovine *S. agalactiae* mastitis remains largely indirect (24, 26). Tulathromycin and gamithromycin are included mainly as long-acting veterinary macrolides that provide pharmacological and PK/PD context from bovine respiratory disease studies rather than direct mastitis evidence (19, 27, 28). Therefore, inclusion in this review indicates scientific relevance, not interchangeability, approval, or proven suitability for lactating dairy mastitis therapy.

This review therefore adopts the broader framework of “veterinary macrolides.” First, these drugs share a class-level antibacterial mechanism, namely binding to the 50S ribosomal subunit and inhibiting protein synthesis, which underlies both their antimicrobial activity and their major resistance pathways, especially ribosomal modification and efflux (24). Second, they were all developed primarily for veterinary use, particularly in cattle, and their practical value often depends as much on tissue distribution and persistence as on *in vitro* activity (19, 26, 28). Third, the literature on *S. agalactiae* is unevenly distributed. Restricting the discussion only to drugs with relatively abundant direct data would exclude important pharmacological context, whereas grouping all of them under the label of “tylosin-type” agents would risk overstating their structural relatedness. This broader framework also reflects how these drugs are positioned in actual veterinary practice. Tylosin, tilmicosin, tylvalosin, tildipirosin, tulathromycin, and gamithromycin are not interchangeable compounds, nor should they be treated as such. However, they repeatedly appear in the same therapeutic discussions because they combine the microbiological properties of the macrolide class with long-acting or tissue-oriented pharmacokinetic behavior. This combination is highly relevant when discussing infections in large animals, especially in situations where direct pathogen-specific data are limited and clinicians may be inclined to extrapolate from class experience (20, 29).

For the purposes of this review, the link between veterinary macrolides and *S. agalactiae* is not primarily based on label indications. Most of the better-known injectable veterinary macrolides were developed and studied mainly for respiratory disease rather than mastitis (19, 26–28). Their relevance to *S. agalactiae* arises from a different combination of factors: activity against Gram-positive bacteria, a shared macrolide resistance ecology, and pharmacokinetic properties that raise legitimate questions about tissue exposure and therapeutic reach.

Among the veterinary macrolides, tylosin has the clearest direct link to bovine *S. agalactiae* in the mastitis literature. Entorf et al. (22) comparatively investigated erythromycin and tylosin susceptibility in bovine mastitis streptococci, including 101 *S. agalactiae* isolates, and found good overall agreement between the two macrolides. They also showed that resistant isolates usually carried recognizable macrolide resistance determinants, most commonly *erm(B)*. This study is important because it anchors the discussion in actual bovine *S. agalactiae* isolates rather than in general macrolide theory.

At the same time, pharmacokinetic observations in lactating animals help explain why veterinary macrolides continue to attract attention in mastitis-related discussions. Avci and Elmas (30) demonstrated in healthy Holstein cows that, after parenteral administration, both tylosin and tilmicosin reached higher concentrations in milk than in serum, with tilmicosin showing particularly marked milk exposure. Similar findings have also been reported for tildipirosin in lactating dairy goats, in which the drug persisted far longer in milk and somatic cells than in plasma (31). These findings do not establish clinical efficacy against bovine *S. agalactiae* mastitis, but they do help explain why systemically administered veterinary macrolides remain pharmacologically relevant when intramammary infection is under consideration.

**Figure 3:**
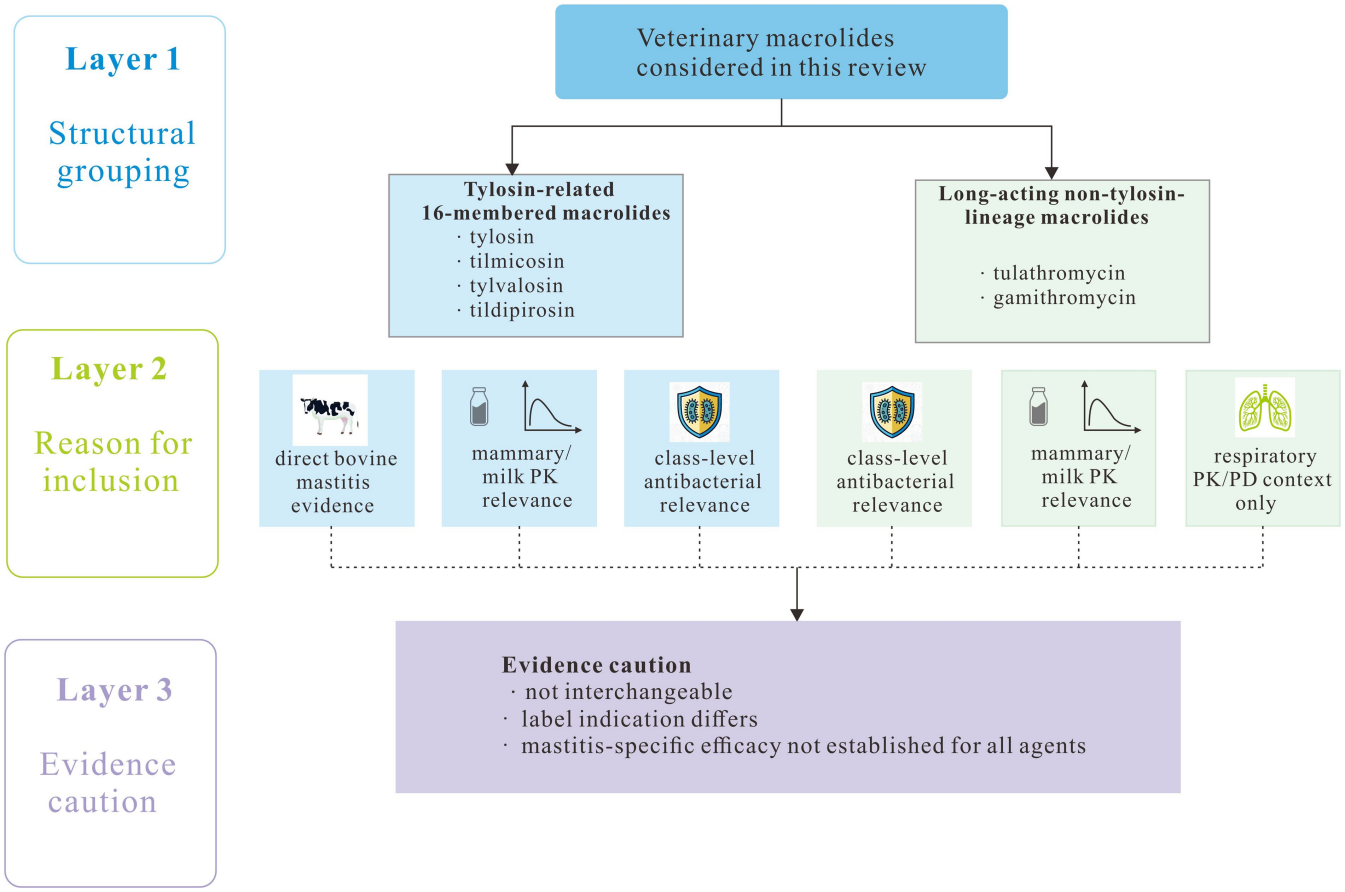
Structural grouping and evidence-based review scope of veterinary macrolides relevant to bovine *S. agalactiae* mastitis (inclusion in this review indicates scientific relevance to antibacterial activity, resistance ecology, or mammary-site exposure, but does not imply approval or suitability for lactating dairy mastitis therapy).

Taken together, among the drugs discussed in this review, tylosin, tilmicosin, and tylvalosin form the core 16-membered macrolide group; tildipirosin strengthens the tylosin-related branch of the discussion; and tulathromycin and gamithromycin broaden the framework to include long-acting veterinary macrolides (Figure 3), thereby providing the foundation for the subsequent discussion of antibacterial basis, resistance, and PK/PD implications.

However, defining the pharmacological scope of veterinary macrolides is not the same as defining their practical availability for lactating dairy mastitis therapy. The inclusion of tylosin, tilmicosin, tylvalosin, tildipirosin, tulathromycin, and gamithromycin in this review should not be interpreted as implying that all of these agents are approved, recommended, or practically suitable for treatment of lactating dairy cows with *S. agalactiae* mastitis. A drug may show *in vitro* activity against susceptible streptococci or display prolonged exposure in tissues or milk-related compartments, yet still be unsuitable for routine mastitis therapy because of label restrictions, residue concerns, milk withdrawal requirements, or insufficient pathogen-specific efficacy evidence (20, 24–31).

This distinction is important because several long-acting veterinary macrolides discussed here were developed and evaluated mainly for respiratory disease or other non-mammary indications rather than for intramammary infection. Tulathromycin, gamithromycin, tildipirosin, and tilmicosin are frequently discussed in veterinary pharmacology because of their long persistence, tissue distribution, and use in food-producing animals, but respiratory or systemic indications should not be treated as evidence of suitability for bovine mastitis (19, 26–28). Similarly, tylosin and tylvalosin remain relevant to the macrolide class and Gram-positive antibacterial activity, but their practical use in dairy cattle depends on the specific product label, jurisdiction, animal class, route of administration, and production status of the treated animal (20, 24, 30).

In lactating dairy cattle, milk residue risk is a major practical constraint. The same pharmacokinetic properties that make macrolides attractive from an exposure perspective may also create residue-management concerns, particularly when drugs persist in milk, mammary secretions, or somatic-cell-associated compartments (30, 31). FDA guidance on extra-label drug use states that, for food-producing animals, directions must include the relevant withdrawal, withholding, or discard time for meat, milk, eggs, or other food products when such use is legally permitted. Similarly, EMA describes maximum residue limits as a food-safety mechanism to ensure that edible products such as meat, milk, and eggs do not contain veterinary medicine residues at levels that may pose a risk to consumers. Therefore, high mammary or milk exposure should be interpreted not only as a possible PK/PD advantage, but also as a residue and regulatory constraint.

Accordingly, this review treats regulatory status, milk residue risk, and withdrawal requirements as part of the evidence framework rather than as secondary practical details. The potential role of veterinary macrolides against bovine *S. agalactiae* mastitis should be evaluated by integrating antibacterial activity, resistance determinants, mammary-site PK/PD, approved label conditions, residue avoidance, and antimicrobial stewardship. This distinction helps prevent microbiological or pharmacological plausibility from being overstated as clinical applicability.

## 4 Why *S. agalactiae* matters in bovine mastitis

### 4.1 Disease burden in dairy herds

The importance of *S. agalactiae* in veterinary medicine is most evident in the control of bovine mastitis. Although it has long been regarded as a “classical” contagious mastitis pathogen, field investigations in recent years indicate that this organism continues to persist in dairy production systems and should not be regarded merely as a historical problem (1, 2, 11). This is observed not only in regions where control systems remain underdeveloped, but also in dairy systems where surveillance and herd-level intervention measures have already been established.

In the Emilia-Romagna region of northern Italy, Tamba et al. (32) analyzed 17,056 bulk-tank milk culture results collected from 2,831 dairy herds between 2019 and 2021. Their results showed that even under an active regional control program, the herd-level apparent prevalence declined only from 8.9 to 5.2%, while the annual incidence fell from 3.0 to 1.5%. In addition, herds infected with group B Streptococcus consistently exhibited higher bulk-tank somatic cell counts, with an average increase of approximately 77,000 cells/mL, and their risk of producing non-marketable milk because somatic cell count (SCC) exceeded the legal threshold was nearly three times that of uninfected herds. These quantitative findings indicate that, even when herd-level prevalence declines under an active control program, *S. agalactiae* can still exert measurable effects on herd productivity and raw milk quality, as reflected by increased bulk-tank SCC and higher risk of producing non-marketable milk.

Recent isolate-based studies have reached similar conclusions. In central Thailand, Wataradee et al. (4) isolated and analyzed 100 *S. agalactiae* strains from 58 cows with clinical and subclinical mastitis across 13 dairy herds, while Liu et al. (5) systematically characterized 140 isolates collected from 12 farms in 6 provinces of northern China. Together, these studies show that *S. agalactiae* continues to circulate in modern dairy production systems and is involved in both clinical and subclinical mastitis. Importantly, the impact of this pathogen is not limited to overt clinical cases. Its more significant effects include persistent intramammary infection, elevated somatic cell counts, impaired milk quality, and sustained within-herd transmission, all of which contribute to long-term production losses (3, 33).

### 4.2 Virulence traits related to intramammary infection

One of the main reasons why *S. agalactiae* remains important in bovine mastitis is its strong capacity for mammary colonization and persistent infection. Recent studies on bovine isolates indicate that the virulence-associated gene profile of this organism is relatively stable and is mainly related to adhesion, invasion, and colonization (34, 35).

In bovine mastitis isolates from Thailand, Wataradee et al. (13) found that all strains carried the *bibA, fbsB, and cfb* genes, and that most strains also carried *fbsA* and *cyl*. In the northern China isolates reported by Liu et al. (5), nearly all strains carried *fbsA, cfb, hylB, bibA,* and *cylE*, whereas *gapC, dltA*, and *lac IV* were detected in all tested isolates. Taken together, these findings indicate that bovine *S. agalactiae* commonly retains a set of functional genes closely associated with host adhesion, adaptation to the mammary environment, and establishment of infection. This is also an important biological basis for its progression from colonization to intramammary infection. Studies from other countries support the same view. Zastempowska et al. (36) analyzed 68 bovine mastitis isolates from Poland and found that all strains carried *cfb, cspA, hylB*, and *sip*, while 89.7% displayed a *β*-hemolytic phenotype. Notably, this study also suggested that bovine *S. agalactiae* isolates are not phenotypically uniform. Cytotoxicity varied among strains, and not all isolates showed the same biological behavior *in vitro*. This suggests that *S. agalactiae* should not be simply classified as either a “high-virulence” or “low-virulence” pathogen. More accurately, bovine isolates appear to share a relatively conserved genetic basis related to colonization and infection, while still displaying a certain degree of biological heterogeneity, which may further influence persistence, host interaction, and clinical expression (34, 35, 37, 38).

In addition, Wataradee et al. (13) did not find any significant association between individual virulence genes and either clinical or subclinical mastitis. This finding suggests that field disease phenotypes cannot be explained solely by the presence or absence of a single virulence gene. Host factors, management conditions, infection duration, and bacterial load may be just as important as virulence genes themselves, and may even determine the final disease outcome to a considerable extent.

### 4.3 Biofilm formation and infection-site biology

Biofilm formation provides another biological layer linking mammary colonization, persistence, and incomplete therapeutic response in bovine *S. agalactiae* mastitis. Although *S. agalactiae* is classically discussed as a contagious intramammary pathogen, biofilm-associated growth may allow bacterial populations to persist on mammary epithelial surfaces, in milk-associated matrices, or within protected microenvironments in which antimicrobial exposure and host clearance are reduced (37). This is relevant because persistent infection and elevated somatic cell count may reflect not only bacterial presence but also the ability of certain isolates to maintain infection-site niches over time.

This biology has direct implications for antimicrobial interpretation. Standard MIC testing is performed against planktonic bacteria and therefore may underestimate the drug exposure required to inhibit or eradicate biofilm-associated populations. A macrolide-susceptible MIC phenotype should not be interpreted as equivalent to activity against bacteria embedded in biofilm or protected within mammary inflammatory microenvironments. Biofilm growth may alter local pH, bacterial metabolic activity, matrix binding, and drug penetration, thereby shifting the apparent exposure-response relationship.

For veterinary macrolides, the implication is not that biofilm formation necessarily excludes efficacy, but that planktonic susceptibility data is incomplete when used alone. Future studies should therefore combine planktonic MIC testing with minimum biofilm inhibitory concentration (MBIC), minimum biofilm eradication concentration (MBEC), biofilm-disruption assays, mammary epithelial cell-associated models, and bacteriological cure endpoints. Such data would help determine whether macrolide exposure in milk or mammary tissue is sufficient against biofilm-associated *S. agalactiae* rather than only against planktonic bacteria. Biofilm-associated tolerance may also justify future evaluation of macrolide-containing combination strategies, but such use should remain evidence-driven and stewardship-based. Rather than assuming that combination therapy is beneficial, studies should determine whether combinations reduce MBIC or MBEC values, improve biofilm disruption, enhance mammary-site bacterial clearance, or increase pathogen-specific bacteriological cure compared with macrolide monotherapy.

### 4.4 Treatment and control of *S. agalactiae*

If *S. agalactiae* were simply a Gram-positive pathogen that remained broadly susceptible to commonly used antimicrobials and depended strictly on contagious transmission, its control would be relatively straightforward. In reality, however, field conditions are clearly more complex. First, this organism has a strong capacity for persistence. Molecular epidemiological work by Wataradee et al. (4) showed that specific *S. agalactiae* strains could persist within the same dairy herd for 2 to 12 months. Long-term strain persistence increases the risk of repeated herd-level exposure and sustained transmission, making eradication more difficult, especially when infected quarters are missed or transmission during milking is not effectively interrupted. Second, the ecological niche of bovine *S. agalactiae* may be broader than traditionally assumed. Cobo-Ángel et al. (7) detected this organism not only in milk samples but also in fecal and environmental samples from Colombian dairy herds, challenging the traditional view that *S. agalactiae* is always a strictly intramammary pathogen. Barsi et al. (39) reported an outbreak caused by ST103 in a closed dairy herd in Italy that remained difficult to eliminate despite conventional control measures, suggesting that environmental transmission or extra-mammary persistence may contribute to infection maintenance in some herds. These studies do not negate the contagious nature of *S. agalactiae*, but they do indicate that control failure cannot be attributed solely to transmission during milking. In some herds, extra-mammary reservoirs or environmental circulation may also help sustain infection (3, 9, 10, 40).

Third, *in vitro* susceptibility does not necessarily mean that field eradication will be easy. Recent studies from Thailand and China indicate that bovine *S. agalactiae* remains broadly susceptible to several *β*-lactam agents, but resistance profiles to other antimicrobial classes vary substantially by region, and some populations have already developed macrolide- and lincosamide-associated resistance phenotypes and resistance genes (4, 5). More importantly, herd-level problems caused by *S. agalactiae* cannot be solved simply by choosing a drug that appears active *in vitro* (14–17). Effective control also depends on timely identification of all infected animals or infected quarters, interruption of transmission, and prevention of reintroduction or persistence of the pathogen at the herd level.

**Figure 4:**
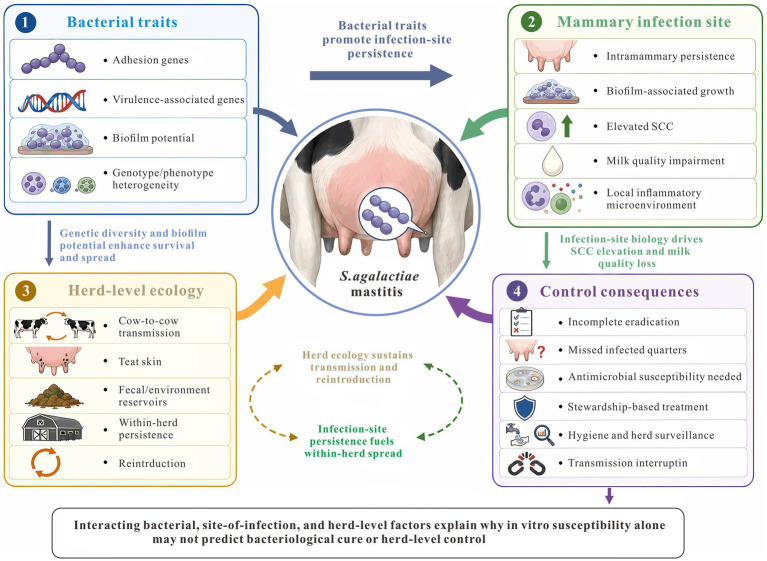
Biological, infection-site and herd-level factors underlying the importance of *S. agalactiae* in bovine mastitis. SCC is somatic cell count.

Therefore, *S. agalactiae* remains important in veterinary medicine, not only because it is a well-recognized mastitis pathogen in its own right, but also because it lies at the intersection of microbiology, herd epidemiology, and antimicrobial stewardship. Its pathogenicity and transmission are supported by a relatively clear biological basis, yet their practical control remains distinctly complex. This is the fundamental reason why the organism continues to warrant sustained attention. The major biological and herd-level factors influencing the continued circulation of *S. agalactiae* and the effectiveness of clinical control are summarized in Figure 4.

## 5 Antibacterial basis of veterinary macrolides against *S. agalactiae*

### 5.1 Ribosomal target and inhibition of protein translation

**Figure 5:**
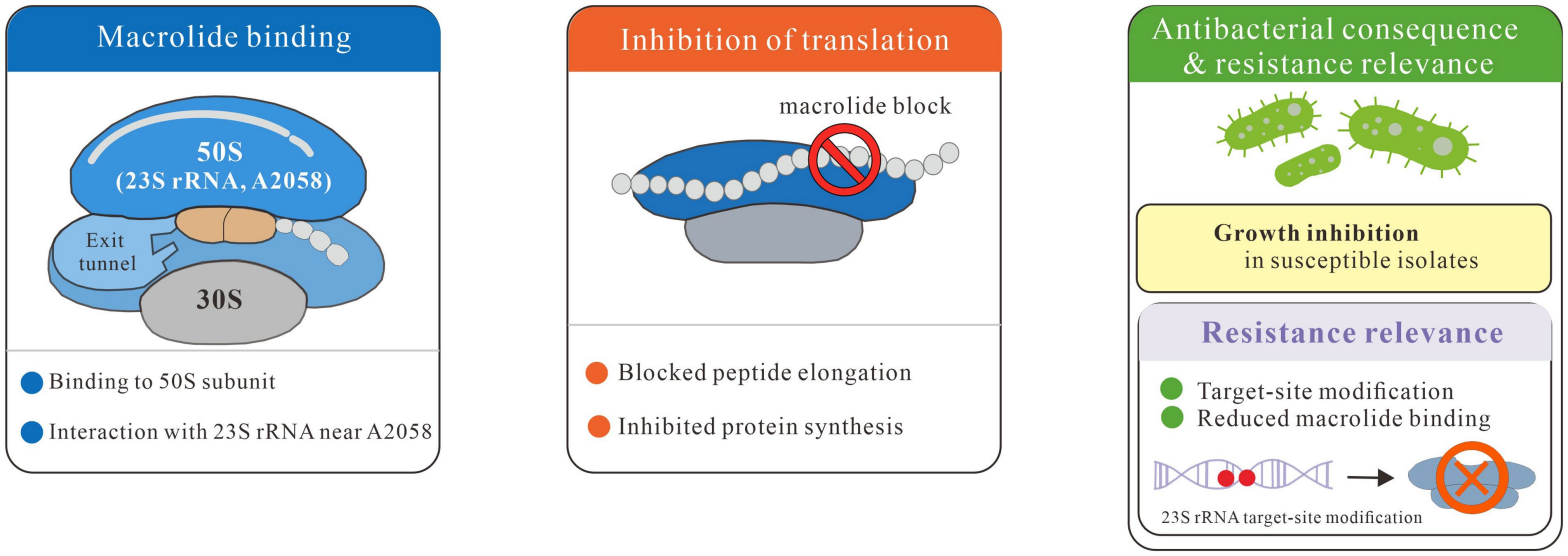
Ribosome-targeting mechanism of veterinary macrolides against *S. agalactiae.*

The antibacterial basis of veterinary macrolides against *S. agalactiae* is first grounded in the well-defined class mechanism shared by these agents. Macrolides exert their activity by binding to the large bacterial ribosomal subunit, with the binding site located in the nascent peptide exit tunnel adjacent to the peptidyl transferase center. Structural studies have shown that 16-membered macrolides, represented by tylosin, can occupy this tunnel and thereby interfere with peptide elongation. At the same time, key nucleotide residues in 23S rRNA, especially A2058 and neighboring sites in the *Escherichia coli* numbering system, constitute an essential structural basis for drug binding. It is precisely this conserved ribosome-targeting mechanism that explains why susceptible *S. agalactiae* isolates can, in principle, be inhibited by veterinary macrolides, and also why drug activity declines rapidly once target-site modification occurs. In other words, this antibacterial basis is not merely hypothetical, but is rooted in the shared ribosomal biology of the macrolide class (41, 42). The ribosome-targeting mechanism of macrolides against *S. agalactiae*, together with the effect of target-site modification on drug binding and translational inhibition, is summarized in Figure 5.

However, in the context of this review, a clearly defined mechanism of action does not necessarily equate to demonstrated clinical efficacy. Ribosomal binding only indicates that veterinary macrolides are biologically plausible agents against *S. agalactiae*. Whether this plausibility can truly translate into practical effectiveness in mastitis control depends on two additional conditions: first, the resistance background of bovine *S. agalactiae* populations; and second, whether the drug can achieve and maintain effective exposure at the site of intramammary infection. It is particularly important to note that, at present, the evidence for macrolides against *S. agalactiae* is much stronger at the microbiological level than at the disease-outcome level, a point that is especially important for the discussion that follows (20, 29).

### 5.2 Direct *in vitro* evidence in bovine isolates

Available direct microbiological evidence indicates that veterinary macrolides still retain measurable antibacterial activity against a proportion of bovine *S. agalactiae* isolates, although this activity is not consistent across studies or isolate populations. Although the French survey conducted by Guérin-Faublée et al. (43) included only a limited number of *S. agalactiae* isolates, it already suggested the presence of macrolide and/or lincosamide resistance among mastitis-associated streptococci. Subsequently, Denamiel et al. (44) analyzed the antimicrobial susceptibility of 36 *S. agalactiae* isolates from bovine intramammary infections in Argentina and reported an erythromycin MIC_90_ of 8.0 μg/mL, while no resistance to penicillin G was detected. In addition, constitutive MLSB and M phenotypes were identified among erythromycin-resistant streptococci. Taken together, these findings suggest that the response of bovine *S. agalactiae* to macrolides has shown clear heterogeneity from an early stage and cannot be simply described as “uniformly susceptible” (14–17).

The evidence most directly related to tylosin comes from the study by Entorf et al. (22). That study compared the susceptibility of 303 bovine mastitis streptococcal isolates to erythromycin and tylosin, including 101 *S. agalactiae* isolates. The results showed a high overall concordance between erythromycin and tylosin susceptibility testing, and almost all erythromycin-resistant isolates carried at least one macrolide resistance determinant, with *erm(B)* being the most common. The importance of this study lies in the fact that it grounds the discussion in bovine mastitis streptococci and directly includes tylosin-based data, rather than relying solely on class-level extrapolation from human-use macrolides such as erythromycin or azithromycin.

More recent studies on bovine isolates further suggest that macrolide activity is still retained in many *S. agalactiae* populations, although not at a uniformly high level of susceptibility. In Thailand, Wataradee et al. (4) reported that most isolates were susceptible to erythromycin by broth microdilution, and in their 2024 follow-up study, 99% of 100 bovine isolates remained susceptible to erythromycin (13). Liu et al. (5) reported an erythromycin susceptibility rate of 89.3% among 140 *S. agalactiae* isolates from northern China. By contrast, De Oliveira et al. (45), in a long-term analysis of bovine *S. agalactiae* isolates from Brazil collected between 1987 and 2021, found that 44 of 156 isolates were erythromycin-resistant. Together, these data indicate that macrolides can retain activity against a substantial proportion of bovine *S. agalactiae* isolates, but reported erythromycin susceptibility or resistance varies markedly across regions, periods, and isolate collections, ranging from high susceptibility in recent Thai and Chinese studies to 44 erythromycin-resistant isolates among 156 Brazilian isolates collected over a long-term period (4, 5, 13, 45).

This heterogeneity is also reflected in the relationship between resistance phenotype and genotype. Liu et al. (5) found that macrolide resistance genes were not uncommon among bovine isolates, with *erm(B)* detected in as many as 75.0%. However, genotype and phenotype were not fully concordant, indicating that the presence of a resistance determinant does not always translate directly into an observed resistant phenotype. This incomplete concordance deserves attention because it suggests that *in vitro* susceptibility results support the antibacterial potential of macrolides, but they cannot replace a more detailed analysis of the resistance structure within the target population (5, 15, 16, 18, 46).

Although these in vitro findings are important, they should be interpreted within the limits of available veterinary breakpoints. Reported MIC values and susceptibility percentages provide microbiological evidence of macrolide activity against selected bovine *S. agalactiae* populations, but they do not by themselves establish clinical susceptibility in bovine mastitis (5, 22, 44, 46–50). In particular, when mastitis-specific clinical breakpoints are unavailable or unclear for a given macrolide–pathogen combination, MIC data should be interpreted descriptively and should be linked to resistance genotype, mammary-site exposure, regulatory feasibility, and bacteriological outcome data where available (20, 30, 31, 51, 52).

### 5.3 Supportive evidence from dry-cow and clinical studies

Compared with *in vitro* susceptibility studies, direct clinical evidence for veterinary macrolides against *S. agalactiae* is clearly more limited and also more difficult to interpret. On the one hand, many mastitis treatment trials enrolled mixed Gram-positive intramammary infections rather than focusing specifically on *S. agalactiae*. On the other hand, some dry-cow or prepartum studies mainly examined overall cure rates or postpartum mastitis occurrence, and often lacked sufficient statistical power after stratification by pathogen. As a result, these studies provide useful reference information, but they are difficult to regard as decisive evidence (53, 54).

The study by Mohammadsadegh et al. (55) is a representative example. It compared intramammary infusion of tilmicosin with cloxacillin during the dry period. Although the overall bacteriological cure rate in the tilmicosin group was lower than that of the control treatment, the more relevant point for the present review lies in the pathogen-specific findings: tilmicosin showed no therapeutic effect against *S. agalactiae*, whereas it demonstrated stronger activity against *Staphylococcus aureus*. This is an important negative result and should be retained explicitly in the review, because it helps prevent the discussion of macrolides from becoming overly optimistic.

Abani et al. (56) evaluated the systemic use of tylosin and tilmicosin in dry Holstein cows 3 weeks before calving, in animals with subclinical mastitis caused by *Staphylococcus aureus* and Streptococcus spp. The results showed that the two macrolide-treated groups had numerically higher overall cure rates than the control group, but these differences did not reach statistical significance after stratification by pathogen. Therefore, this study is better regarded as supportive but insufficient evidence. It suggests that periparturient systemic macrolide treatment may exert some biological effect in the context of mixed mastitis, but it is still insufficient to establish a clear pathogen-specific efficacy signal for *S. agalactiae*.

Broader mastitis treatment studies have provided similar information. McDougall et al. (57) found that in dairy cows with clinical mastitis caused predominantly by Gram-positive pathogens, injectable tylosin achieved clinical and bacteriological cure rates comparable to those of penethamate hydriodide. However, it should be noted that the predominant isolate in that study was *Streptococcus uberis*, and the trial was not specifically designed to evaluate treatment outcomes in *S. agalactiae*. Therefore, while this study supports the view that tylosin has a certain degree of clinical activity in Gram-positive mastitis, it cannot be taken as direct evidence of clear efficacy against bovine *S. agalactiae* mastitis (53, 54, 57).

Taken together, the antibacterial basis of veterinary macrolides against *S. agalactiae* may be summarized as follows: the mechanism is clear, the microbiological evidence is supportive, but the clinical significance remains insufficiently resolved. At the mechanistic level, the picture is relatively clear. Macrolides act on a conserved ribosomal target, and structural studies related to tylosin provide a solid basis for their inhibitory activity against susceptible streptococci. At the microbiological level, available studies on bovine isolates, particularly those directly involving tylosin, show that a substantial proportion of *S. agalactiae* populations remain susceptible to macrolides. What remains unclear, however, is the disease-level meaning of these findings. Existing clinical and dry-cow studies are limited in number, not fully consistent in outcome, and in most cases do not provide sufficiently robust pathogen-specific analyses for *S. agalactiae*, so they are still insufficient to support strong therapeutic conclusions.

Accordingly, veterinary macrolides have a credible antibacterial basis against susceptible *S. agalactiae* isolates, but their practical value remains highly dependent on the resistance epidemiology of the target population and the PK/PD conditions at the site of infection. These two issues are precisely the focus of the following sections.

## 6 Resistance determinants and susceptibility interpretation

Macrolide resistance in *S. agalactiae* should not be interpreted as a single phenotype or a single-gene event. In bovine mastitis isolates, the available evidence indicates that *erm*-mediated target-site modification, particularly involving *erm(B)*, represents the most consistently reported mechanism of macrolide resistance (5, 45–47, 58). However, other resistance pathways are also relevant to susceptibility interpretation. Efflux-associated determinants, lincosamide-related genes, inducible resistance phenotypes, mobile genetic elements, and co-selection with tetracycline resistance genes may all influence the relationship among resistance phenotype, genotype, cross-resistance potential, and clinical interpretation (44, 59–63).

### 6.1 Major resistance phenotypes and erm-mediated target-site modification

**Figure 6:**
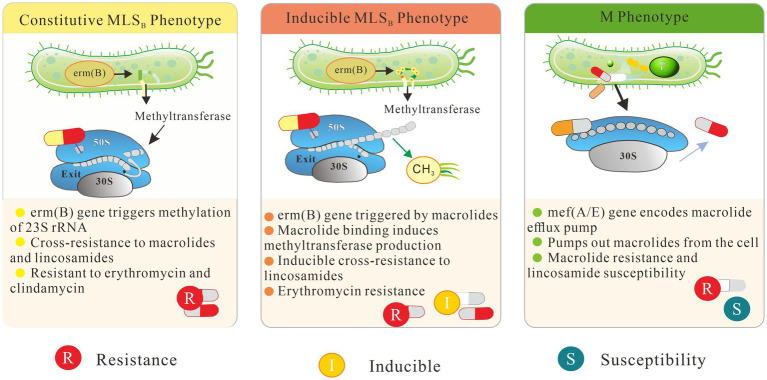
Major macrolide resistance phenotypes in *S. agalactiae.* The cMLSB and iMLSB phenotypes are mainly associated with erm-mediated target-site modification, whereas the M phenotype is usually associated with efflux-mediated resistance involving *mef(A/E)* and related determinants.

In *S. agalactiae*, macrolide resistance is mainly expressed through three phenotypic patterns: constitutive macrolide–lincosamide–streptogramin B resistance (cMLSB), inducible macrolide–lincosamide–streptogramin B resistance (iMLSB), and the M phenotype (44, 59, 62). These phenotypes differ in their mechanisms and clinical implications. The cMLSB and iMLSB phenotypes are usually associated with erm-mediated ribosomal target-site modification and may imply cross-resistance to macrolides, lincosamides, and streptogramin B. By contrast, the M phenotype is generally associated with active macrolide efflux and is typically characterized by macrolide resistance while lincosamide susceptibility may be retained (59, 61, 62). These phenotype-level relationships and their implications for cross-resistance interpretation are summarized in Figure 6.

The distinction between cMLSB and iMLSB resistance is clinically relevant because inducible resistance may be missed if erythromycin and lincosamide susceptibility are interpreted without appropriate induction testing (59, 63). Available bovine data suggest that the cMLSB phenotype is particularly relevant in mastitis-associated *S. agalactiae*. Denamiel et al. (44) reported that erythromycin resistance among bovine mastitis streptococci was mainly associated with the constitutive MLSB phenotype, while the M phenotype occurred in only a smaller subset of isolates and inducible MLSB resistance was not observed in that study. Human *S. agalactiae* populations may show more diverse resistance phenotypes, including M, cMLSB, and iMLSB patterns, indicating that resistance profiles from human group B streptococci should not be directly transferred to bovine mastitis isolates without considering host origin and bacterial population structure (46, 58, 59).

**Figure 7:**
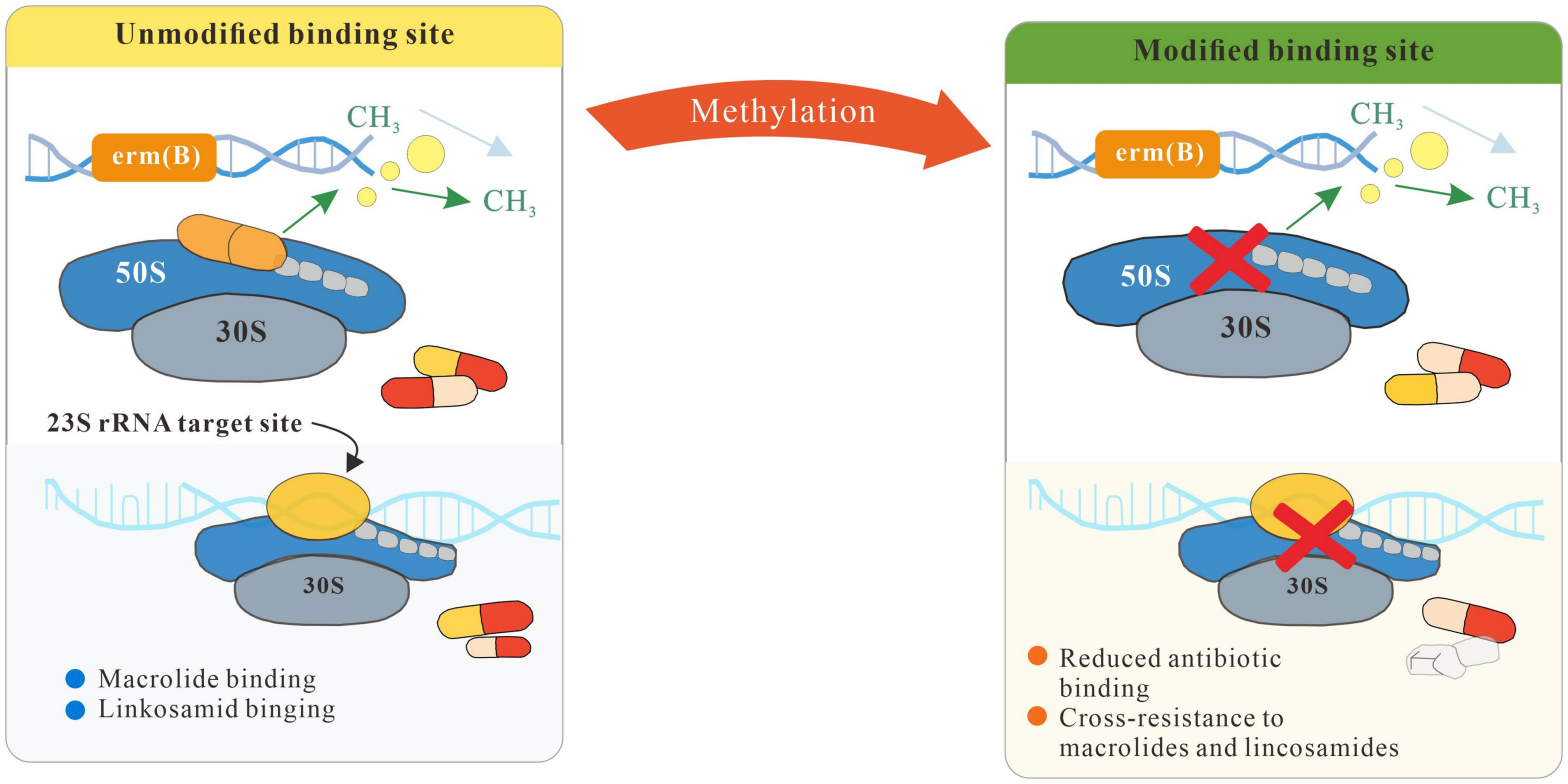
*erm(B)*-mediated target-site modification and reduced macrolide binding in *S. agalactiae*.

At the molecular level, erm genes encode methyltransferases that modify the 23S rRNA target site and reduce macrolide binding to the large ribosomal subunit (41, 42, 62, 63). This mechanism is important because it affects the ribosomal binding site shared by macrolides, lincosamides, and streptogramin B. The effect of *erm(B)*-mediated target-site modification on macrolide binding is illustrated in Figure 7. Several studies support the central role of *erm(B)* in bovine *S. agalactiae*. Dogan et al. (47) found that *erm(B)* was the only erythromycin resistance determinant detected among resistant bovine isolates in their comparative bovine–human dataset. Gao et al. (46) identified *erm(B)* as one of the common resistance genes in bovine mastitis isolates in China. More recently, Liu et al. (5) reported that among 140 bovine *S. agalactiae* isolates from clinical mastitis cases in northern China, *erm(B)* was detected in 75.0% of isolates, a frequency much higher than that of *erm(A)*, *erm(C)*, *erm(TR)*, or *mef(A)*. These findings support the conclusion that *erm(B)* is currently the most important macrolide resistance determinant in many bovine *S. agalactiae* populations.

However, *erm(B)* dominance should not be interpreted as exclusivity. Other erm variants, including *erm(A)* and *erm(TR)*, remain relevant because they may be associated with inducible or constitutive MLSB phenotypes and may occur in mobile genetic backgrounds (60, 63). Mingoia et al. (60) showed that *erm(TR)* in *S. agalactiae* can be located in different integrative and transposable elements, indicating that less frequent *erm* determinants may still have dissemination potential. Thus, *erm(B)* should remain the central focus in bovine isolates, but a broader erm-associated resistance ecology should also be acknowledged.

### 6.2 Supplementary resistance mechanisms, mobile elements, and co-selection

Efflux-mediated resistance should be considered when interpreting macrolide susceptibility, even though it appears less consistently reported in bovine *S. agalactiae* than erm-mediated resistance. The *mef(A/E)* genes encode macrolide efflux systems and are usually associated with the M phenotype (59, 61, 62). The *msr(D)* gene is frequently linked to *mef*-containing resistance modules and may contribute to macrolide resistance through efflux-associated or ribosomal protection-like mechanisms (61, 62). In bovine mastitis isolates, *mef(A/E)* and *msr(D)* currently appear to be less dominant than *erm(B)*; for example, Liu et al. (5) did not detect *mef(A)* in their bovine isolates from northern China. Nevertheless, efflux-related mechanisms should remain within the interpretive framework, particularly when the observed phenotype resembles the M pattern or when genotype–phenotype relationships cannot be fully explained by *erm* genes alone (59, 61, 62).

Lincosamide-related determinants also deserve brief consideration because macrolide resistance interpretation is often linked to lincosamide susceptibility and MLSB cross-resistance. The *lnu* genes encode lincosamide nucleotidyltransferases that inactivate lincosamides, whereas *lsa*-type genes are generally associated with ABC-F family resistance mechanisms and may contribute to lincosamide-, streptogramin A-, or pleuromutilin-associated resistance (64–66). Achard et al. (64) identified *lnu*(C) in *S. agalactiae*, and Malbruny et al. (65) reported cross-resistance associated with *lsa*(C) in this species. More recent genomic evidence also indicates that *lsa*(E) and *lnu*(B) can occur in group B Streptococcus within mobile resistance contexts (66). Direct evidence for these genes in bovine *S. agalactiae* remains more limited than that for *erm(B)*, so they should be regarded as supplementary determinants rather than major drivers of bovine macrolide resistance.

Mobile genetic elements provide an additional layer of interpretation. Genes such as *erm*, mef, msr, *lnu*, *lsa*, and tetracycline resistance genes may be embedded in transposons, integrative and conjugative elements, or other mobile genetic platforms (60, 61, 63, 66). This is relevant because tetracycline resistance is common in many bovine *S. agalactiae* populations (5, 15, 17, 45, 58). Genes such as *tet(M)* and *tet(O)* encode ribosomal protection proteins and are often associated with mobile elements in Gram-positive bacteria (58, 62, 63). When tetracycline resistance determinants coexist with macrolide or MLSB resistance genes on related mobile platforms, antimicrobial use in one class may help maintain resistance to another class through co-selection. Therefore, macrolide resistance should be interpreted within a broader multidrug-resistance ecology rather than as an isolated phenotype.

### 6.3 Genotype–phenotype discordance and breakpoint limitations

Although molecular detection of resistance determinants is useful, genotype does not always predict phenotype perfectly in bovine *S. agalactiae*. Gao et al. (46) noted that some isolates carried resistance genes despite being phenotypically susceptible, whereas some phenotypically resistant isolates lacked the expected detected genes. Liu et al. (5) reported a similar pattern in bovine isolates from northern China, where many isolates carried macrolide resistance genes without showing corresponding phenotypic resistance. Such discordance may reflect differences in gene expression, promoter activity, inducibility, gene integrity, regulatory effects, susceptibility testing methods, breakpoint selection, or resistance mechanisms not included in the detection panel (5, 46, 63). Therefore, molecular testing should not replace phenotypic susceptibility testing. Instead, MIC or disk diffusion results and resistance gene profiles should be interpreted together.

Breakpoint interpretation further complicates susceptibility assessment. Veterinary breakpoints are most informative when they are established for a defined pathogen–host–drug–disease combination and supported by microbiological, PK/PD, dosage-regimen, and clinical outcome data (48–50). CLSI VET01S provides evidence-based tables for disk diffusion and dilution susceptibility testing of bacteria isolated from animals, but the availability of a veterinary AST standard does not mean that validated clinical breakpoints exist for every pathogen–drug–host–infection-site combination (48). For several macrolides discussed in this review, especially long-acting agents developed mainly for respiratory disease, bovine mastitis-specific clinical breakpoints for *S. agalactiae* are limited or unclear (19, 26–28, 48, 67–69). Therefore, interpretive criteria from other pathogens, host species, infection sites, or routes of administration should not be transferred automatically to bovine *S. agalactiae* mastitis.

EUCAST/VetCAST MIC distributions and epidemiological cut-off values can help identify wild-type and non-wild-type populations, but epidemiological cut-off values (ECOFFs) should not be treated as clinical breakpoints (49, 50). This distinction is important in mastitis because clinical outcome depends not only on MIC, but also on mammary-site drug exposure, the free active fraction, inflammation, milk and tissue distribution, withdrawal requirements, and pathogen-specific bacteriological cure (30, 31, 51, 52). In the bovine *S. agalactiae* literature, macrolide susceptibility has often been interpreted using available veterinary criteria, erythromycin as a class reference, study-specific MIC distributions, or genotype–phenotype comparisons (5, 22, 44, 46, 47). These approaches provide useful microbiological information, but they should be regarded as surrogate interpretive strategies rather than definitive proof of clinical susceptibility.

Accordingly, this review treats MIC values and susceptibility percentages primarily as microbiological evidence rather than direct evidence of clinical efficacy. A low MIC or susceptible phenotype indicates *in vitro* responsiveness, but it does not prove bacteriological cure in the bovine udder. Conversely, a resistant phenotype, especially when associated with *erm(B)* or another MLSB-related determinant, should be considered a warning sign for reduced macrolide or lincosamide usefulness (5, 44–47, 58). When validated bovine mastitis-specific macrolide breakpoints are unavailable, isolates should be described more cautiously as having low or high MICs, wild-type or non-wild-type distributions when ECOFFs are available, or phenotypic susceptibility according to the criteria used in the original study (48–50).

### 6.4 Implications for susceptibility interpretation and treatment decisions

The practical implication of these resistance mechanisms is that macrolide susceptibility in bovine *S. agalactiae* should be interpreted conservatively. When *erm(B)* or another erm determinant is present, macrolide resistance should not be treated as the isolated loss of activity of a single drug; it may indicate broader MLSB cross-resistance, especially when the cMLSB or iMLSB phenotype is observed (5, 44, 46, 58). When the M phenotype is present, efflux-associated mechanisms such as *mef(A/E)* and *msr(D)* should be considered, and any retained lincosamide activity should be evaluated by actual susceptibility testing rather than assumed (59, 61, 63). When *lnu*, *lsa*, *tet(M)*, *tet(O)*, or mobile resistance elements are present, the result should be interpreted in the broader context of multidrug-resistance ecology and antimicrobial stewardship (48, 58, 63, 66).

For bovine mastitis, the most defensible approach is to integrate resistance phenotype, resistance genotype, MIC or disk diffusion results, mammary-site pharmacological feasibility, breakpoint limitations, approved label status, withdrawal requirements, and available bacteriological outcome data. *In vitro* susceptibility alone does not establish clinical efficacy, and gene detection alone does not fully define the phenotype (5, 46, 58). To clarify the relationship among resistance determinants, mechanisms, phenotypes, cross-resistance patterns, and clinical interpretation, the major resistance determinants relevant to macrolide and MLSB resistance in bovine *S. agalactiae* are summarized in Table 1.

**Table 1:** Major resistance determinants associated with macrolide and MLSB resistance in bovine *S. agalactiae*.

Resistance determinant	Main mechanism	Typical phenotype	Affected antimicrobial classes	Cross-resistance implication	Relevance to bovine *S. agalactiae*	Representative evidence
*erm(B)*	23S rRNA methylation and reduced macrolide binding to the ribosome	cMLSB or iMLSB	Macrolides, lincosamides, streptogramin B	High risk of MLSB cross-resistance	Most consistently reported and most important macrolide resistance determinant in many bovine isolates	(5, 45–47, 58)
*erm(A)*/*erm(TR)*	23S rRNA methylation; may be inducible or constitutive depending on regulatory context	iMLSB or cMLSB	Macrolides, lincosamides, streptogramin B	Cross-resistance possible, especially when MLSB phenotype is expressed	Less frequent than *erm(B)* in bovine isolates, but relevant because of mobility and dissemination potential	(47, 60, 62, 63)
*mef(A/E)*	Active macrolide efflux	M phenotype	Mainly macrolides, especially 14- and 15-membered macrolides	Lincosamide activity may be retained, depending on actual susceptibility results	Less consistently detected in bovine isolates than *erm(B)*, but important when M phenotype or erm-negative macrolide resistance is observed	(59, 61–63)
*msr(D)*	Efflux-associated or ribosomal protection-like contribution, often linked to mef-associated modules	Usually associated with efflux-type macrolide resistance	Macrolides	Supports interpretation of non-erm macrolide resistance	Direct bovine *S. agalactiae* evidence is more limited, but it should be considered in broader resistance surveillance	(61–63)
*lnu* genes	Lincosamide nucleotidyltransferase-mediated drug inactivation	Lincosamide resistance	Lincosamides	May complicate interpretation of MLSB-related treatment options	Supplementary mechanism; relevant when considering lincosamide alternatives or cross-resistance patterns	(64, 66)
*lsa* genes	ABC-F family resistance or ribosomal protection-like mechanism	Lincosamide-, streptogramin A-, or pleuromutilin-associated resistance	Mainly lincosamide-related classes, depending on gene variant and host background	May contribute to multidrug resistance interpretation	Limited direct evidence in bovine *S. agalactiae*; should be interpreted cautiously as part of broader MLSB ecology	(65, 66)
*tet(M)*, *tet(O)* linked elements	Ribosomal protection against tetracyclines; often associated with mobile genetic platforms	Tetracycline resistance, with possible co-selection of other resistance genes	Tetracyclines; indirect relevance to MLSB resistance through co-selection	Co-maintenance of tetracycline and macrolide/MLSB resistance determinants may occur when genes are linked on mobile elements	Relevant because tetracycline resistance is common in bovine *S. agalactiae* and may influence herd-level resistance ecology	(5, 15, 17, 45, 58, 62, 63)
Transposons and integrative conjugative elements	Horizontal transfer and genetic linkage of resistance determinants	Multidrug resistance or lineage-associated resistance profiles	Depends on carried resistance genes	Facilitate dissemination and co-selection of resistance determinants	Important for explaining persistence, spread, and regional differences in resistance profiles	(60–63, 66)

cMLSB, constitutive macrolide–lincosamide–streptogramin B resistance; iMLSB, inducible MLSB resistance; M phenotype, efflux-associated macrolide resistance phenotype.

The clinical meaning of each determinant should be interpreted together with MIC results, induction testing where app ropriate, resistance gene detection, approved label status, milk withdrawal requirements, and antimicrobial stewardship considerations.

## 7 Mammary-specific PK/PD implications

### 7.1 Distribution, persistence and tissue exposure

From a pharmacokinetic perspective, the continued interest in veterinary macrolides largely stems from their generally large apparent volumes of distribution, long elimination half-lives, and marked tissue accumulation. Using gamithromycin as an example, Huang et al. (19) showed in cattle that, after intravenous administration, the steady-state volume of distribution was approximately 24.9 L/kg, and bioavailability after subcutaneous injection was nearly complete. More importantly, drug exposure in lung tissue was far higher than in plasma, with lung-to-plasma concentration ratios reaching 265, 410, 329, and 247 at 1, 5, 10, and 15 days after administration, respectively, and the lung tissue area under the concentration–time curve (AUC) being approximately 194-fold higher than that of plasma. Similarly, Menge et al. (26) reported that tildipirosin concentrations in bovine bronchial fluid remained at a plateau of approximately 3.5 μg/g from 1 to 3 days after administration, while the half-lives in lung tissue and bronchial fluid were about 10 and 11 days, respectively. Taken together, these findings indicate that the main advantage of long-acting veterinary macrolides does not lie primarily in “high plasma concentrations,” but rather in their ability to rapidly enter target tissues and persist there for prolonged periods (20, 21, 29).

For mastitis research, the behavior of these drugs in milk and mammary secretions is of even greater interest. Avci and Elmas (30) found in healthy Holstein cows that after intramuscular administration of tylosin, the C_max_ in milk was 4.55 μg/mL, whereas that in serum was only 1.30 μg/mL; after subcutaneous administration of tilmicosin, the milk C_max_ was 20.16 μg/mL, compared with 0.86 μg/mL in serum. Correspondingly, the AUC_𝑚𝑖𝑙𝑘_/AUC_serum_ ratios were 5.01 and 23.91, respectively, indicating a clear tendency for both drugs to distribute into milk, with tilmicosin showing particularly pronounced milk exposure. Mendoza et al. (51) further reported that in dry cows, subcutaneous administration of tilmicosin resulted in peak concentrations of 14.4–20.9 μg/mL in mammary secretions. Galecio et al. (31) observed a similar pattern in lactating goats, where the half-life of tildipirosin in milk was approximately 58.3 h, much longer than the 6.2 h observed in plasma; when the somatic cell fraction was also considered, exposure was even higher than in plasma. For *S. agalactiae* mastitis, these quantitative PK findings indicate that some veterinary macrolides can achieve substantially higher and more persistent exposure in milk or mammary-related compartments than in plasma; however, such total concentration data still require cautious interpretation because they do not directly define free active drug exposure or bacteriological efficacy at the mammary infection site.

### 7.2 PK/PD indices relevant to macrolides interpretation

For macrolides, AUC/MIC is often considered an important PK/PD index because many agents in this class show exposure-dependent activity combined with prolonged persistence. However, in mastitis, the relevant index should ideally be based on free, biologically active drug exposure at the mammary infection site rather than total plasma or total milk concentration. Depending on the compound, bacterial population, and infection model, free AUC/MIC, C_max_/MIC, and time above MIC may all contribute to interpretation, but none of these indices has been sufficiently validated for bovine *S. agalactiae* mastitis. This is particularly relevant because the milk/serum AUC ratios reported for tylosin and tilmicosin were 5.01 and 23.91, respectively, yet these total exposure ratios cannot by themselves determine the free AUC/MIC target required for bacteriological cure in bovine *S. agalactiae* mastitis (30).

The same MIC value may therefore have different therapeutic meaning depending on whether the drug exposure is measured in plasma, milk, mammary secretion, somatic cells, mammary interstitial fluid, or inflamed tissue. For long-acting macrolides, prolonged persistence can support exposure over time, but the active fraction available to interact with bacteria may be lower than the total concentration measured analytically. This distinction is especially important when interpreting high milk concentrations as evidence of therapeutic potential.

### 7.3 Milk pH, ion trapping, protein binding, and inflammation

Mammary PK/PD interpretation is further complicated by the physicochemical behavior of macrolides. As weak bases, macrolides may undergo pH-dependent distribution and ion trapping between plasma, milk, inflammatory exudate, and cellular compartments. Milk pH can change during mastitis, and inflammation can alter the permeability of the blood-milk barrier, the cellular content of milk, and the composition of mammary secretions. These changes may increase total drug entry into mammary compartments while still leaving uncertainty about the free drug concentration at the bacterial microenvironment.

Milk fat, milk protein, casein binding, and cellular association may also cause total milk concentration to differ from free antimicrobial exposure. Therefore, high milk or secretion concentrations should be interpreted as evidence of mammary distribution, not as direct proof of bacteriological efficacy. This distinction becomes even more important when biofilm-associated populations or intracellular-like protected niches are present.

Because total plasma or milk concentrations do not necessarily represent active drug exposure at the mammary infection site, key mammary PK/PD considerations for selected veterinary macrolides are summarized in Table 2.

**Table 2:** Mammary pharmacokinetic and PK/PD considerations relevant to veterinary macrolides in bovine mastitis.

Macrolide	Main evidence source	Mammary or milk exposure feature	Key PK/PD implication	Interpretation caveat	Representative evidence
Tylosin	Healthy lactating cows; mastitis-related clinical studies	Milk concentrations may exceed serum concentrations after parenteral administration	Supports mammary distribution and biological plausibility	Healthy-cow milk exposure cannot define mastitis-specific efficacy	(30, 57)
Tilmicosin	Healthy cows, dry cows, mammary secretion studies	Marked milk or mammary secretion exposure	Potential relevance to intramammary infection	Residue constraints and pathogen-specific outcome uncertainty remain important	(30, 51, 55)
Tylvalosin	Veterinary macrolide class evidence; limited mastitis-specific data	Limited direct mammary PK evidence	Mechanistically relevant as tylosin-related 16-membered macrolide	Direct bovine *S. agalactiae* mastitis evidence is insufficient	(24)
Tildipirosin	Lactating goat milk and somatic cell data; cattle respiratory PK	Prolonged persistence in milk and somatic cell fraction reported in goats	Suggests compartmental accumulation and long tissue persistence	Goat and respiratory data cannot be directly extrapolated to bovine mastitis	(26, 31)
Tulathromycin	Cattle respiratory PK/PD and tissue distribution studies	Strong tissue distribution, mainly lung/PELF data	Useful for class-level PK/PD principles	Does not establish mammary-site exposure or mastitis efficacy	(27, 28, 52)
Gamithromycin	Cattle respiratory PK/PD studies	Large volume of distribution and high tissue exposure	Supports long-acting macrolide exposure framework	Evidence mainly from respiratory disease models	(19, 21, 49, 67)

PELF, pulmonary epithelial lining fluid.

Total milk or tissue concentration should not be equated with free, biologically active drug concentration at the mammary infection site.

### 7.4 Limits of extrapolating respiratory PK/PD models to mastitis

Most of the existing PK/PD evidence for veterinary macrolides was not established in the context of mastitis, but rather derives from respiratory infection models, pulmonary epithelial lining fluid (PELF), bronchial fluid, or serum-based systems. In naturally occurring bovine respiratory disease, DeDonder et al. (67) found that higher PK/PD indices were generally associated with better treatment outcomes; for *Pasteurella multocida* in particular, the AUC_0–24_ /MIC in PELF was significantly associated with therapeutic success. Similarly, Zeng et al. (70) established a PK/PD model for tildipirosin in a murine lung infection model of *Pasteurella multocida*, while Wang et al. (68) reported in a *Streptococcus suis* model that the AUC_24h_/MIC of gamithromycin was most closely associated with antibacterial efficacy. They also emphasized, however, that their study addressed only serum-based PK/PD relationships and that the true PK/PD target values corresponding to drug concentrations at the infection site still required further validation. In other words, although AUC/MIC as a predictive index for long-acting macrolides is reasonably well supported, that support comes mainly from respiratory pathogens or non-mammary models rather than from *S. agalactiae* mastitis itself (21, 67, 69).

This point is methodologically crucial. Foster et al. (52), after comparing drug concentrations in calf pulmonary epithelial lining fluid, interstitial fluid, and plasma, pointed out that drug levels at the actual site of infection are critical for predicting therapeutic efficacy, whereas plasma, tissue homogenates, or certain surrogate compartments do not necessarily provide an accurate representation of target-site exposure. The same principle applies when this concept is transferred to mastitis. PELF-AUC/MIC values, lung tissue persistence, or bronchial fluid plateau concentrations that are useful in respiratory disease cannot be directly equated with effective exposure in milk, mammary interstitial fluid, or intramammary infection foci. Thus, respiratory PK/PD models provide useful class-level context for veterinary macrolides, but they do not define mammary-site exposure targets or bacteriological efficacy thresholds for *S. agalactiae* mastitis.

### 7.5 Evidence gaps specific to intramammary infection

Although the currently available mammary-related PK evidence indicates that macrolides can enter milk and mammary secretions, this remains far from establishing PK/PD targets for *S. agalactiae* mastitis. There are at least three reasons for this. First, many studies have been conducted in healthy cows, dry cows, or even lactating goats, and the resulting parameters may not fully represent drug behavior under clinical mastitis conditions. Second, most existing studies remain limited to drug concentrations in milk or secretions and lack systematic integration with pathogen MIC distributions, resistance genotypes, and bacteriological clearance outcomes. Third, total drug concentration in milk does not necessarily equal the effective drug exposure at the actual site of infection. This is particularly relevant for agents with strong tissue and cellular accumulation, in which differences among milk, somatic cells, mammary interstitial fluid, and the lesion microenvironment may all influence the final interpretation of efficacy. Accordingly, the available evidence suggests that veterinary macrolides have the potential to achieve high exposure in mammary-related compartments, but the quantitative relationship between such exposure and bacteriological cure of *S. agalactiae* mastitis has not yet been established. This also helps explain why drugs with similarly high concentrations in mammary secretions do not always yield consistent conclusions in clinical studies. What is currently lacking is research that truly integrates drug concentrations at the mammary infection site with *S. agalactiae* MIC distributions, resistance profiles, and bacteriological clearance rates (30, 31, 51, 69).

### 7.6 Implications for rational use and antimicrobial stewardship

**Figure 8:**
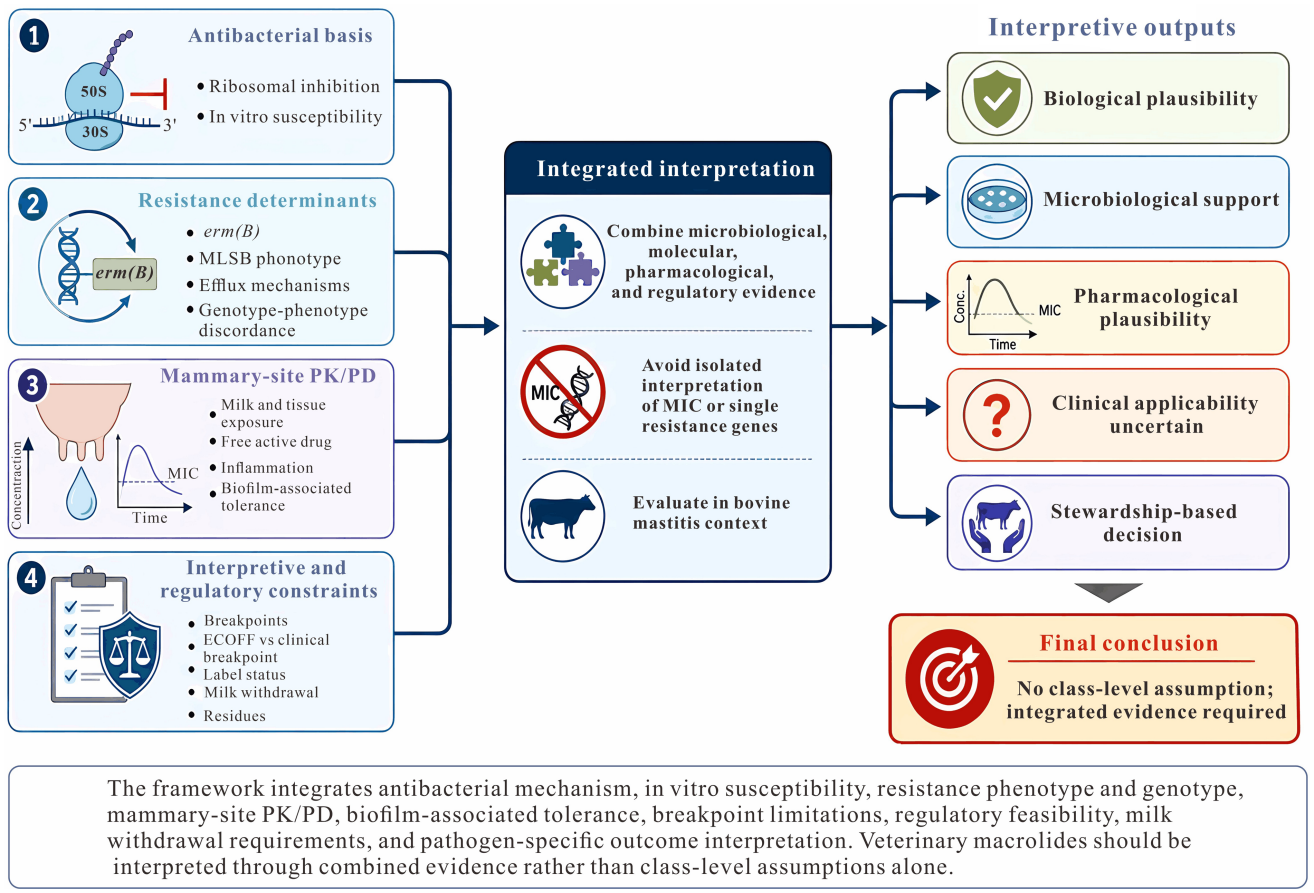
Integrated framework for interpreting veterinary macrolides against bovine *S. agalactiae* mastitis.

Based on the above analysis, the PK/PD implications of veterinary macrolides in *S. agalactiae* can be summarized in two points. First, macrolides possess noteworthy mammary-related exposure characteristics, which provide a pharmacological basis for further evaluation in intramammary infection. Second, practical interpretation must return to a pathogen–site–host framework: the MIC distribution and resistance background of bovine *S. agalactiae* should be considered together with free or biologically active drug exposure at the mammary infection site, local inflammatory conditions, regulatory feasibility, and bacteriological outcomes. This integrated interpretation is summarized in Figure 8.

For clinical application and antimicrobial stewardship, veterinary macrolides should not be regarded as automatically effective options on the basis of class experience or favorable tissue penetration alone. Their suitability should be evaluated using isolate-level susceptibility testing, resistance genotype, mammary-site exposure, withdrawal or residue-management requirements, and pathogen-specific bacteriological outcomes (20, 29, 58). This is especially relevant for drugs with prolonged milk persistence, for which pharmacological value and practical constraints may coexist. Overall, the PK/PD evidence supports continued investigation of veterinary macrolides in bovine *S. agalactiae* mastitis, but does not yet provide a mature basis for routine therapeutic recommendations.

## 8 Stewardship and diagnostic application

### 8.1 Culture and susceptibility testing as the foundation

For bovine mastitis caused by *S. agalactiae*, antimicrobial stewardship should begin with pathogen identification and isolate-level susceptibility testing. Regional surveillance data can guide empirical expectations, but they cannot replace herd- or isolate-level testing because macrolide susceptibility and resistance determinants vary by geography, lineage, and herd history. This is particularly important for macrolides because the same class-level mechanism of action can be undermined by target-site modification, efflux, or linked resistance elements.

### 8.2 Molecular detection of resistance determinants

Rapid molecular detection of *erm*, *mef*, *msr*, and selected lincosamide- or tetracycline-associated genes may provide useful early information before macrolide use, especially when clinical decisions must be made before full phenotypic results are available. Detection of *erm(B)*, in particular, should alert veterinarians to a high risk of MLSB-associated cross-resistance. However, molecular detection should not be used as the sole determinant of treatment because genotype–phenotype discordance has been reported in bovine *S. agalactiae*. Molecular testing is therefore best regarded as a stewardship adjunct rather than a replacement for culture and phenotypic susceptibility testing.

### 8.3 Practical decision framework

A practical decision framework should integrate five elements: pathogen confirmation, MIC or susceptibility phenotype, resistance genotype, mammary-site exposure, and regulatory feasibility. Macrolide use should be considered unsupported when MLSB-associated resistance is detected, when bovine mastitis-specific breakpoints are unavailable and MIC interpretation is uncertain, when mammary-site PK/PD evidence is insufficient, or when label restrictions and milk withdrawal requirements make use impractical. In this sense, stewardship does not simply mean reducing macrolide use; it means restricting use to situations in which microbiological, pharmacological, regulatory, and clinical evidence align.

## 9 Current research gaps and challenges

Despite the growing body of literature on *S. agalactiae* in dairy cattle, important gaps remain in how existing evidence is interpreted and connected. The problem is no longer simply the absence of data. Studies have already described antimicrobial susceptibility, resistance genes, virulence factors, molecular epidemiology, and selected pharmacokinetic features. However, these lines of evidence are still rarely integrated within a unified framework. As a result, current knowledge remains stronger at the level of isolate description than at the level of clinical interpretation. Key questions therefore remain insufficiently answered, including which bovine strains are more likely to persist, which resistance patterns are more likely to compromise treatment, and which genetic or phenotypic characteristics are truly associated with bacteriological or clinical outcome (3, 11, 40).

One major challenge is the limited linkage between genotype, phenotype, and therapeutic relevance. In bovine *S. agalactiae*, macrolide resistance determinants such as *erm(B)* have been repeatedly identified as important, yet genotype and phenotype do not always show complete concordance. This means that neither resistance gene detection alone nor *in vitro* MIC results alone can adequately predict treatment outcome. Future studies should therefore move beyond descriptive surveillance and adopt a more integrated design, in which susceptibility phenotypes, resistance and virulence genotypes, infection duration, treatment regimens, bacteriological clearance, and SCC dynamics are evaluated together within the same strain set or herd-level cohort. Without such linkage, resistance determinants remain informative at the molecular level but difficult to translate into practical treatment decisions (5, 15, 58).

Another major gap lies in PK/PD interpretation. Current knowledge clearly shows that veterinary macrolides often display extensive tissue distribution and prolonged persistence, making them pharmacologically attractive. However, most established PK/PD frameworks have been derived from respiratory pathogens, lung tissue, or pulmonary epithelial lining fluid models rather than from bovine mastitis caused by *S. agalactiae*. Consequently, much less is known about what level of exposure is actually required at the mammary infection site to achieve bacteriological clearance. Milk or mammary secretion concentrations alone are not sufficient to answer this question, particularly for drugs with strong tissue and cellular accumulation. A priority for future work is therefore the development of mammary site-specific PK/PD models that integrate local drug exposure, isolate MIC distributions, resistance backgrounds, and bacteriological cure data, rather than relying primarily on extrapolation from respiratory disease models (20, 52, 69).

Challenges also remain at the epidemiological level. Available studies indicate that bovine *S. agalactiae* can persist within herds, continue circulating despite control efforts, and display heterogeneous resistance profiles across regions and lineages. Yet most surveillance still relies on single time-point or single-region datasets. Future molecular epidemiological studies should move beyond simple herd-level detection and incorporate whole-genome sequencing, lineage tracking, and longitudinal sampling across broader temporal and geographic scales. Such approaches will be necessary to determine which lineages are more likely to persist, which are more prone to accumulate resistance, and which may be associated with host adaptation or environmental maintenance. Addressing these gaps is essential if the field is to move from documenting the presence of *S. agalactiae* toward understanding its transmission dynamics and improving both therapeutic decision-making and antimicrobial stewardship (2, 9, 10, 71). The major limitations of the current evidence base and recommended future research priorities are summarized in Table 3.

**Table 3:** Key evidence gaps and recommended research priorities for veterinary macrolides against bovine *S. agalactiae* mastitis.

Evidence gap	Current limitation	Why it matters	Recommended future direction
Mastitis-specific breakpoints	Specific veterinary clinical breakpoints may be unavailable for some macrolide–pathogen combinations	MIC values cannot be directly translated into therapeutic decisions	Develop bovine mastitis-specific interpretive criteria or validated surrogate frameworks
Genotype–phenotype linkage	*erm(B)* and other genes do not always correspond perfectly with phenotypic resistance	Gene detection alone cannot replace susceptibility testing	Pair MIC testing with resistance gene detection and expression analysis
Mammary-site PK/PD targets	Most PK/PD targets are derived from respiratory or non-mammary models	Respiratory AUC/MIC targets may not apply to intramammary infection	Establish mammary-site PK/PD models using bovine mastitis isolates
Free drug exposure	Most studies report total milk or secretion concentrations	Total concentration may overestimate active exposure	Measure free drug in milk, mammary tissue, and inflammatory secretions
Biofilm-associated tolerance	Planktonic MIC predominates in current evidence	Biofilm growth may reduce antimicrobial activity	Include MBIC/MBEC and biofilm disruption assays
Pathogen-specific clinical efficacy	Existing dry-cow or mastitis studies often include mixed pathogens and do not provide sufficiently powered *S. agalactiae*-specific cure estimates	Microbiological plausibility cannot be translated into therapeutic recommendations without quantitative bacteriological outcome data	Conduct controlled field studies reporting pathogen-specific bacteriological cure, SCC change, recurrence, and relapse outcomes
Regulatory feasibility	Label restrictions and withdrawal periods are not consistently integrated	Practical use depends on residue and stewardship constraints	Compare approved indications, milk withdrawal, and extra-label limitations by region

## 10 Conclusion

Veterinary macrolides retain measurable antibacterial activity against a substantial proportion of bovine *S. agalactiae* isolates, although resistance determinants such as *erm(B)* and heterogeneous MLSB phenotypes are prevalent. Pharmacokinetic evidence indicates that several macrolides can achieve prolonged mammary-site exposure, but direct pathogen-specific clinical efficacy data for veterinary macrolides against bovine *S. agalactiae* mastitis remain limited. Therefore, current evidence should be interpreted as supporting biological and pharmacological plausibility rather than proven clinical efficacy, especially in the absence of validated mastitis-specific breakpoints, consistent genotype–phenotype concordance, and pathogen-stratified bacteriological outcome data. Regulatory feasibility, milk withdrawal requirements, and antimicrobial stewardship considerations further limit routine use in lactating dairy cattle. Biofilm formation and site-specific tolerance may additionally affect therapeutic efficacy, highlighting the importance of molecular diagnostics to guide treatment decisions.

Future evaluation of veterinary macrolides against bovine *S. agalactiae* mastitis should not rely on class-level assumptions. Instead, it should integrate isolate-level MICs, resistance genotypes, MLSB phenotypes, mammary-site free drug exposure, biofilm-associated tolerance, regulatory feasibility, milk withdrawal requirements, and pathogen-specific bacteriological outcomes. Only through this integrated evidence framework can the therapeutic role of veterinary macrolides be defined in a manner that is scientifically defensible, clinically meaningful, and consistent with antimicrobial stewardship.

## Data Availability

The original contributions presented in the study are included in the article/supplementary material, further inquiries can be directed to the corresponding authors.
